# Vascular Supply of the Human Spiral Ganglion: Novel Three-Dimensional Analysis Using Synchrotron Phase-Contrast Imaging and Histology

**DOI:** 10.1038/s41598-020-62653-0

**Published:** 2020-04-03

**Authors:** Xueshuang Mei, Rudolf Glueckert, Annelies Schrott-Fischer, Hao Li, Hanif M. Ladak, Sumit K. Agrawal, Helge Rask-Andersen

**Affiliations:** 1 Department of Surgical Sciences, Section of Otolaryngology, Uppsala University Hospital, SE 751 85 Uppsala, Sweden; 2 Department of Otolaryngology, Peking University Shenzhen Hospital, Shenzhen, China; 3 Department of Otolaryngology, Medical University of Innsbruck, Anichstr. 35, A-6020 Innsbruck, Austria; 4 Department of Otolaryngology-Head and Neck Surgery, Department of Medical Biophysics and Department of Electrical and Computer Engineering, Western University, London, ON Canada; 5 Department of Otolaryngology-Head and Neck Surgery, Western University, London, ON Canada

**Keywords:** Anatomy, Medical imaging, Medical research

## Abstract

Human spiral ganglion (HSG) cell bodies located in the bony cochlea depend on a rich vascular supply to maintain excitability. These neurons are targeted by cochlear implantation (CI) to treat deafness, and their viability is critical to ensure successful clinical outcomes. The blood supply of the HSG is difficult to study due to its helical structure and encasement in hard bone. The objective of this study was to present the first three-dimensional (3D) reconstruction and analysis of the HSG blood supply using synchrotron radiation phase-contrast imaging (SR-PCI) in combination with histological analyses of archival human cochlear sections. Twenty-six human temporal bones underwent SR-PCI. Data were processed using volume-rendering software, and a representative three-dimensional (3D) model was created to allow visualization of the vascular anatomy. Histologic analysis was used to verify the segmentations. Results revealed that the HSG is supplied by radial vascular twigs which are separate from the rest of the inner ear and encased in bone. Unlike with most organs, the arteries and veins in the human cochlea do not follow the same conduits. There is a dual venous outflow and a modiolar arterial supply. This organization may explain why the HSG may endure even in cases of advanced cochlear pathology.

## Introduction

Human inner ear function relies on microcirculation derived from vessels in the internal auditory canal (IAC). A disrupted vascular supply leads to immediate inner ear changes^1,2^ and may cause several inner ear disorders, such as sudden deafness, vertigo, and vestibular neuritis. Experimentally, numerous drugs have been shown to be effective in increasing cochlear blood flow^3,4^, but there is little evidence of clinical benefit. The advent of inner ear surgeries, such as cochlear implantation (CI), have renewed interest in the vascular anatomy of the human inner ear. Cochlear neurons are targeted in CI, and their retained vitality is required for a sustained hearing outcome. Furthermore, electrode insertion affecting the vascular supply of the cochlea can potentially lead to ganglion cell injury.

The human inner ear is supplied with blood vessels from the labyrinthine end artery which subdivides into three branches. Two branches, the cochlea-vestibular artery (CVA) and the cochlear artery (CA), enter through the fundus region of the IAC. A third branch, the anterior vestibular artery (AVA), supplies the vestibular organ together with the vestibular branch of the CVA^5–7^. The cochlear arteries supply the spiral lamina, limbus, and the lateral cochlear wall tissue responsible for the unique ion composition of endolymph and the endo-cochlear potential (EP) essential for hair cell transduction^8^. The vessels supply the modiolus and spiral ganglion neurons (*n* = 35,000) located in a bony spiral called the Rosenthal canal (RC). The sensory epithelium with hair cells (*n* = 15,000) surprisingly lack vascularization, and their metabolic supply may depend on the surrounding fluids and cells^9^. The CVA divides into cochlear and vestibular branches. The cochlear branch follows the spiral course of the modiolus and anastomoses with the CA^5,6,10^ forming anastomosing arcades with branches of the CA. However, the CA is often missing in certain anatomic variants, and in these cases, the cochlear branch of the CVA supplies the entire cochlea. Both the cochlear and vestibular branches are believed to supply the capillary regions of the human spiral ganglion (HSG).

The venous system is even more complex, and initial reports by Eichler^7^ did not describe a double venous outflow. Later studies reported two separate venous systems, which are located in the scala tympani (ST) and in the scala vestibuli (SV), respectively^5,9^. The veins were named the anterior spiral vein (ASV) and posterior spiral vein (PSV) by Nabeya^5^ and Axelsson^9^ later renamed them the SV and ST veins. The veins form a series of segments that are said to run in opposite directions. Both veins drain segments of the HSG and the external wall. The SV vein is situated centrally in the modiolus, composed of separate segments, and drains the spiral lamina and the SV of the entire cochlea. According to Axelsson^9^, all human cochleae are different with respect to connections between the two venous systems. From the literature, it remains unclear which role the tractus arteriosus spiralis foraminosus (TASF) plays: arterial, venous, or both. It is a prominent channel running on the upper side of the lower basal turn^5^ According to Nabeya, the PSV begins at the junction between the lower two-thirds and the upper one-third of the basal coil and is involved in the drainage of the upper HSG.

Anatomic investigations of human cochlear blood vessels are challenging due to their helical shape and encasement in the hardest bone in the body. Furthermore, there are anatomic variations, extensive interspecies differences, and no general consensus as to the nomenclature of these blood vessels^9^. Previous studies primarily used various injection techniques combined with decalcification, sectioning, clearance, and surface preparations^5–7,9,11^; however, the results of these techniques depend on the ability to completely fill or stain the vascular branches, and three-dimensional (3D) modeling is difficult and susceptible to artifacts^12^. Vogel *et al*.^13^ used absorption micro-tomography with a spatial resolution of approximately 10 μm, but soft tissue structures such as the basilar membrane were not visible within the cochlea. Improvements in visualization may be gained by staining with osmium tetroxide, phosphotungstic acid, or iodine (I_2_) in alcohol-immersion^14^. Lareida *et al*.^15^, with a different synchrotron technique, and Müller *et al*.^16^ used staining with 1% OsO4 to visualize cellular structures, such as the organ of Corti, stria vascularis, membranous labyrinth, nerve fiber bundles, and even subcellular structures in ganglion cells. Although visualization is significantly improved, the process can lead to stain-induced shrinkage of the soft tissues^17^. Rau *et al*.^18^ obviated the use of staining through the use of phase-contrast imaging (PCI) to highlight soft tissues. Their group dissected cochleae from gerbils, cut them along the mid-modiolar plane, and imaged the hemi-cochleae. The basilar membrane and hair cells could be visualized on projections; however, 3D reconstructions and modelling were not presented. Elfarnawany *et al*.^19^ were the first to perform synchrotron radiation phase-contrast imaging (SR-PCI) to examine intact human temporal bones and cochleae, providing excellent soft tissue detail and visualization of cytoarchitecture^20^.

PCI is similar to conventional radiography. It comprises an X-ray source, an examined specimen, and a detector with no other optical elements. The detector is placed at a specified distance from the sample to allow the phase-shifted beam to interfere with the original beam and produce fringes. These fringes correspond to the surfaces and structural boundaries of the sample (edge enhancement), as opposed to a conventional radiogram which does not have these features. While computed tomography (CT) imaging is absorption contrast-based, PCI can potentially be combined with SR-PCI to improve soft-tissue contrast with accurate visualization of bone. In PCI, the phase shift is caused by transformation into detectable variations in X-ray intensity. Previous studies have demonstrated that SR-PCI can be used to visualize both bone and soft tissue simultaneously.

The objective of the present study was to combine light microscopy (LM) with novel SR-PCI to create the first accurate 3D model of the HSG vascular supply. The synchrotron allowed for accurate, high-resolution images without decalcification or sectioning. Phase-contrast was added to highlight soft tissues without the need for contrast agents. The locations of the arterial and venous channels were also assessed for potential damage during CI insertion. A preliminary study combining μCT and synchrotron imaging was recently presented^20^.

## Material and Methods

### Ethics Statement

All human temporal bone specimens used for SR-PCI were obtained with permission from the Body Bequeathal Program at Western University, London, Ontario, Canada in accordance with and approved by the Anatomy Act of Ontario and Western University’s Committee for Cadaveric Use in Research (approval #19062014). For histological analyses, archival sections of the HSG were used^21^. Re-evaluation from archival celloidin-embedded sections emanated from previous research projects and were published by Spoendlin and Schrott^22^. These studies were approved by the University of Innsbruck and the experimental protocols were in accordance with the regulations of the Scientific Committee at the Medical University. Human bodies were donated to the Division of Clinical and Functional Anatomy of the Innsbruck Medical University by individuals who had given their informed consent prior to death for the use of their bodies for scientific and educational purposes^23,24^. The studies adhered to the rules of the Declaration of Helsinki.

#### SR-PCI

The SR-PCI technique used, in-line PCI, was earlier described by Elfarnawany *et al*.^19^ and Koch *et al*.^25^. Furthermore, the materials used in the present study were previously described in studies of the human RC using a combined analysis of μCT and synchrotron imaging performed on adult fresh-frozen cadaveric temporal bones^20,26^. In brief, the 26 human temporal bones were thawed and cut to 40 mm × 60 mm lengths and fixed in 3.7% formaldehyde and 1% glutaraldehyde in a phosphate buffer for five days. The samples were rinsed and dehydrated in graded ethanol. No additional processing (i.e., staining, sectioning, or decalcification) was performed on the samples. Fixation reduced tissue degradation between sample preparation and imaging. Samples were transferred to the imaging facilities in motion-proof containers to prevent damage during shipping.

Each sample was scanned using the BioMedical Imaging and Therapy (BMIT) 05ID-2 beamline at the Canadian Light Source Inc. (CLSI) in Saskatoon, SK, Canada. It provides an SR beam produced by a superconducting wiggler source^27^. The beam was filtered using a customized Si (111) bent double Laue crystal monochromator and yielded an energy bandwidth of ΔE/E = 10^−3^ over an energy range of 20–150 keV^19^. In this study, the monochromator was tuned to achieve an X-ray photon energy of 47 keV for all the scans. After tuning the incident beam angle of the monochromator to meet the Bragg diffraction condition of 47 keV, only the beam at 47 keV (0.263 Å) was allowed through the monochromator for the imaging experiments. The energy resolution ΔE/E was approximately 10^−3^ based on the intrinsic reflection feature of the selected monochromator crystal Si (111) and was also affected by the mechanical bent. With such a small energy bandwidth (approximately ±47 eV), the effect of photon contributions on the acquired images could be ignored. The photon energy of the scan was set at 47 keV to ensure at least 15% X-ray transmission through the specimens which would not significantly reduce the signal-to-noise ratio (SNR)^28^. At the same time, such energy can also maximize image contrast (both absorption and phase contrast) compared with imaging at higher photon energy. The specific value of 47 keV was determined empirically by evaluating image contrast for a number of energy levels.

The imaging setup consisted of a sample stage and a charge-coupled device-based detector system, installed at a beamline length of 57 m from the source. Both were placed on a vibration isolation table. The distance between the sample and detector was 2 m. The detector was coupled with a C9300–124 camera (Hamamatsu Photonics, Shizuoka, Japan) with a 12-bit resolution and pixel size of 9 × 9 µm^2^. The scintillator used in the AA-60 was P43 (Gd2O2S: Tb) with a 10 µm thickness. The detector had an L-shaped quartz optics design for high X-ray radiation tolerance. It included two objectives with 1X magnification. Both lenses were the same (105 mm lens; Pentax, Tokyo, Japan).

The imaging field of view was set to 4,000 × 950 pixels, corresponding to 36.0 × 8.6 mm; 3,000 projections over 180° were acquired per view. The synchrotron CT system uses a parallel beam instead of a cone beam. Therefore, reconstruction was based on the use of a parallel beam. The NRecon (Bruker, Billerica, MA, USA) commercial software was used, which implements the Feldkamp algorithm. NRecon provides algorithms to do both cone beam CT reconstruction and parallel beam reconstruction. In this case, we chose the parallel beam reconstruction mode in NRecon for all data reconstruction. NRecon was chosen over other software because it was a common CT reconstruction program used at the BMIT at the time and provided reconstructed images that were of sufficient quality for further analysis in this study. There is other software that can also perform SR CT reconstruction, even phase retrieval, and we intend to compare the software packages in future work. The resulting 3D image volume had an isotropic voxel size of 9 µm. The acquisition time to capture all projections per scan was ~30 min.

Note that the spatial resolution of the system measured by a resolution phantom was approximately 15 µm. A spatial resolution similar to that in this study can be achieved by laboratory-based µCT techniques. The unique imaging technique used in this study, PCI, provides additional edge-enhanced phase contrast which made the visualization of unstained soft tissue possible. PCI requires a spatially coherent beam which is a feature of a synchrotron beam and is very difficult to achieve using laboratory-based X-ray sources. Also, the high flux and monochromatic beam of synchrotron radiation makes the scan faster and provides better SNR, which are advantages over laboratory-based micro-CT.

With any study of this sort, the potential for radiation damage must be considered. This study focused on 3D visualization of the anatomical microstructure of the human cochlea using fixed specimens. Radiation damage did not affect the anatomical microstructure of the specimens since there was no motion artifact in the reconstructed slices. Hence, we do not discuss potential radiation damage in this paper, but such a study will be conducted in the future.

### Image processing

The large imaging datasets were imported into the open-source medical imaging software, 3D Slicer v.4 (www.slicer.org)^29^. Each sample had approximately 3,000 orthogonal slices through the inner ear, and these were reconstructed in multiple planes to allow for slice-by-slice manual segmentation and verification. Hand segmentation of the vessels was made from 3,000 images over several months. From these samples, a single representative computer-based 3D rendering was created of the RC, which houses the HSG cell bodies. The surrounding bone channels were digitally segmented, modeled, and reconstructed (1552R). To visualize this, the general bone was simply volume rendered, and one bone structure showing the venous outflow pattern was overlaid in color (1552L). Arteries were identified in the IAC, and major veins were traced back from the inferior cochlear vein (ICV). These individual vessels were traced along the HSG; however, small individual bone channels and capillary networks were difficult to visualize on the SR-PCI images. Here, routine LM was used to augment the findings on SR-PCI.

## Results

**Figure 1:**
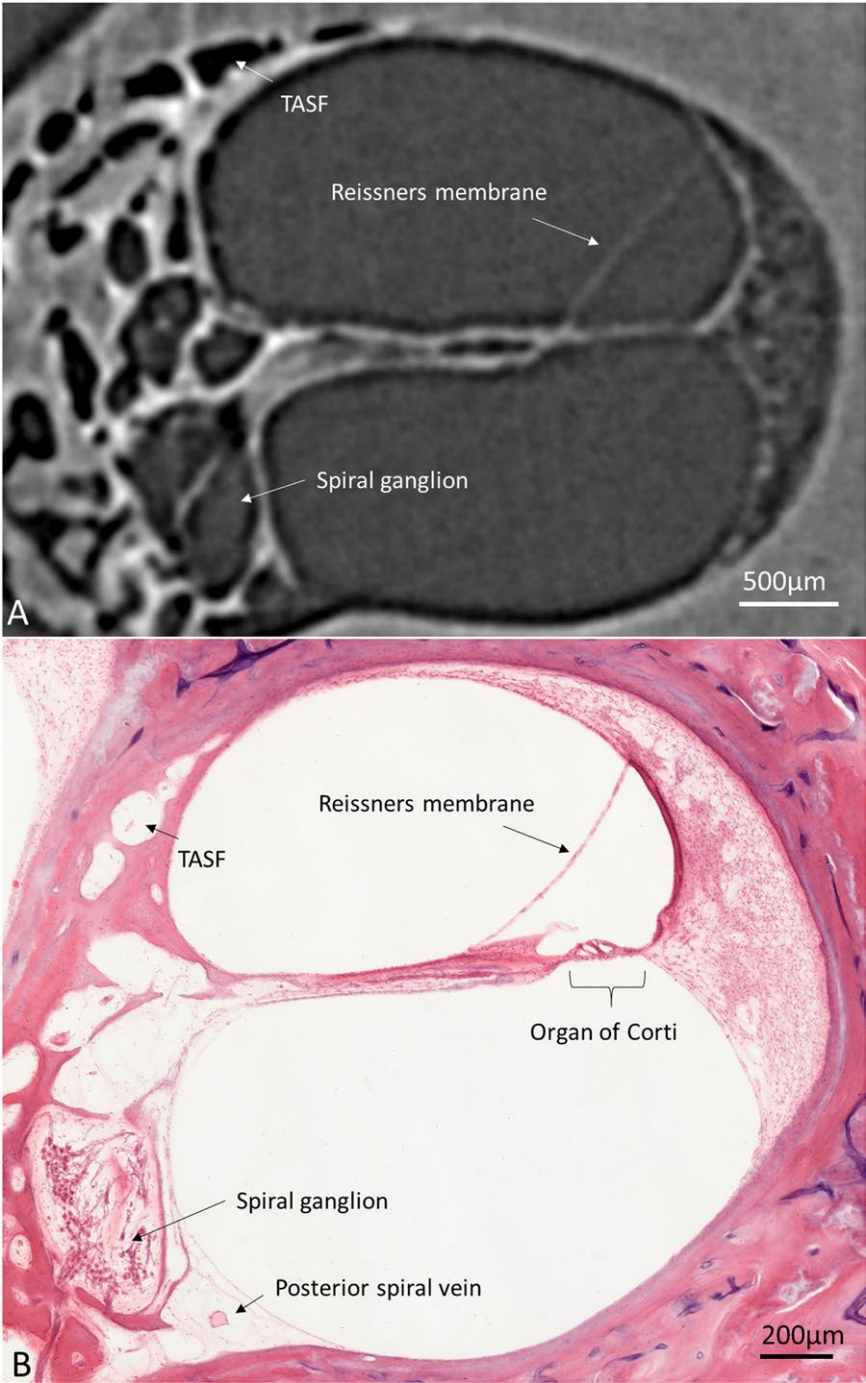
(**A**) Synchrotron section of the basal turn of a human cochlea and a corresponding histological section (**B**) for comparison.

**Figure 2:**
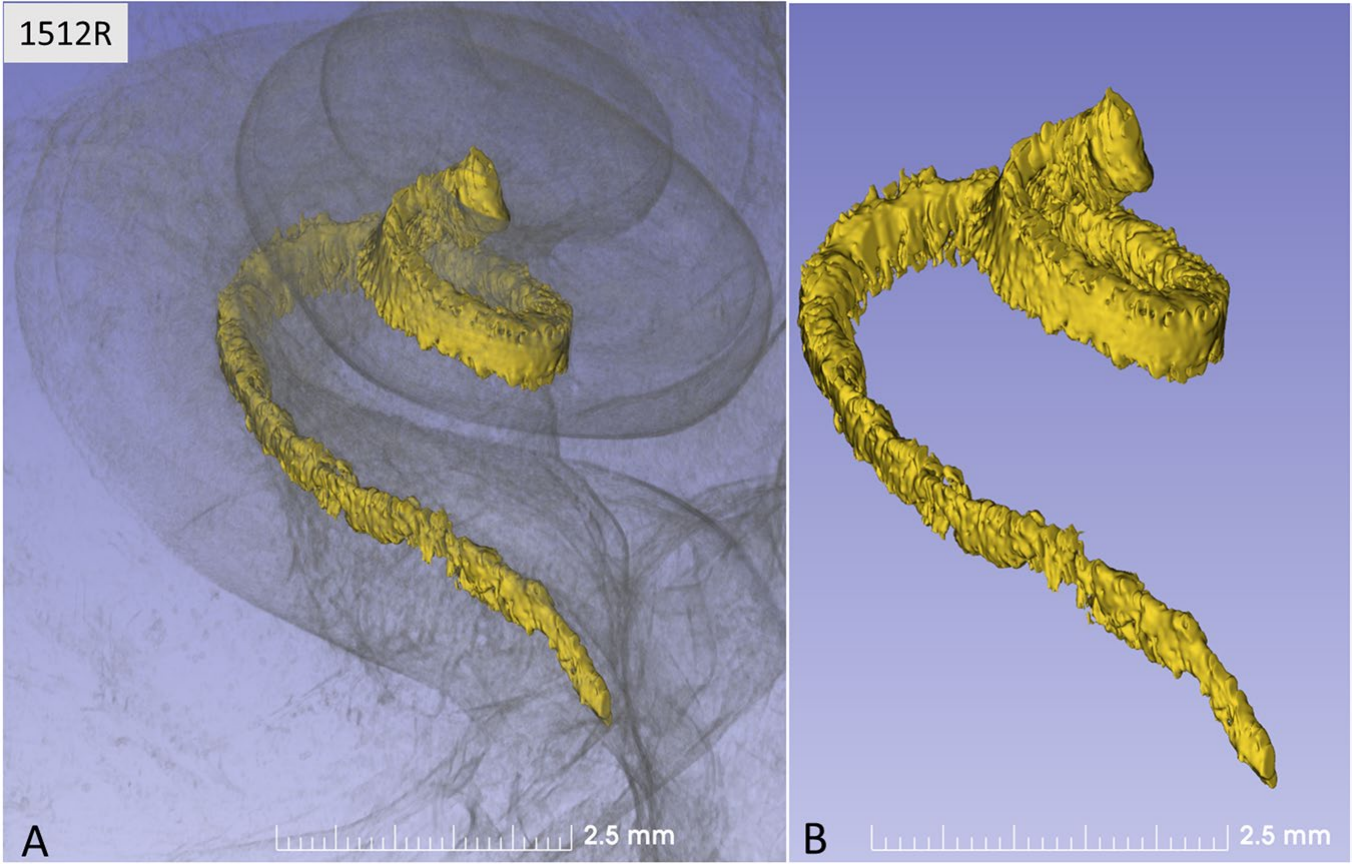
(**A**,**B**) Synchrotron radiation phase-contrast imaging (SR-PCI) and orthographic rendering with 3D view of a left Rosenthal canal (yellow) and its topographic relationship in the semi-transparent cochlea.

**Figure 3:**
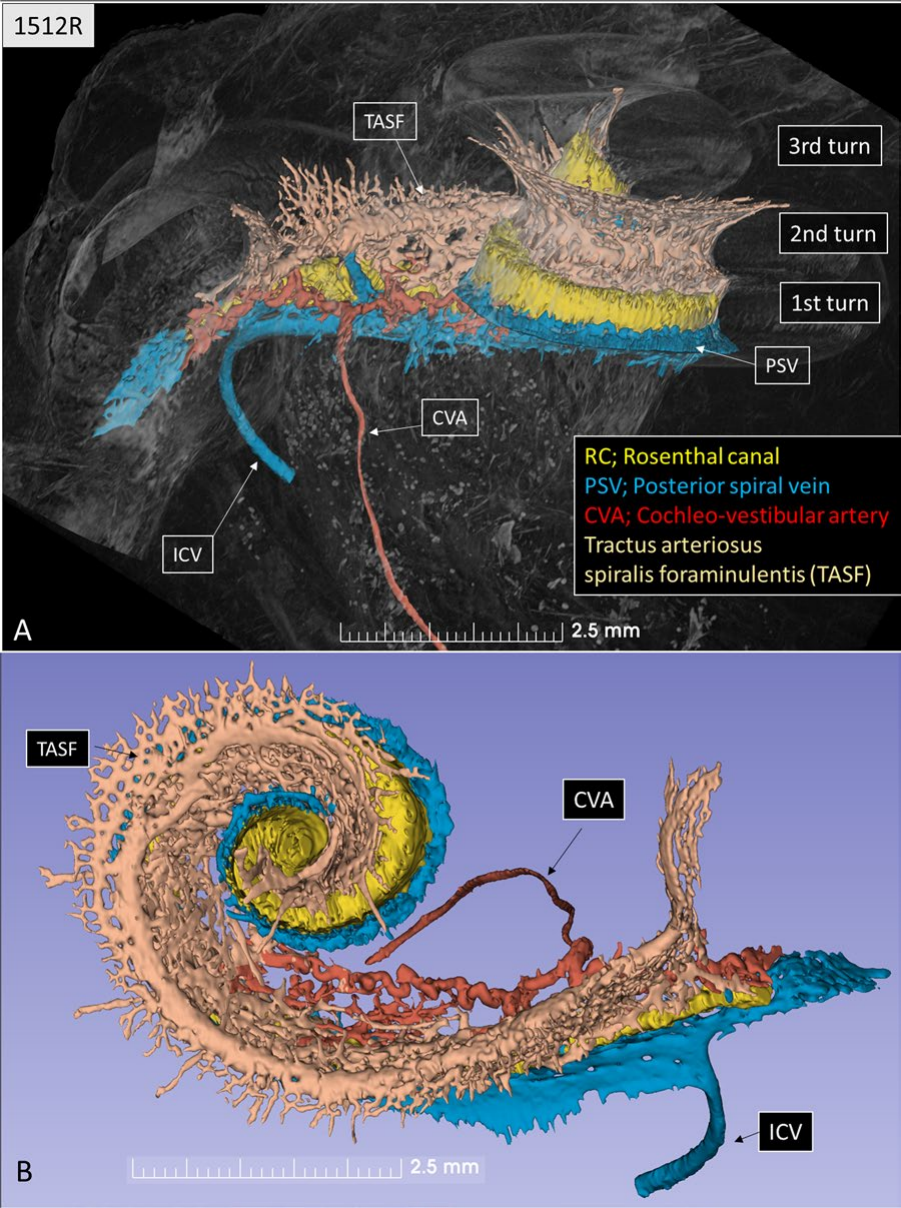
Segmentation and model of the Rosenthal canal (RC), cochlea-vestibular artery (CVA), inferior cochlear vein (ICV), and posterior spiral vein (PSV) channels in a left human cochlea. (**A**) Superior view, (**B**) lateral view. There is a complex bone channel system (brown) named tractus arteriosus spiralis foraminulentis (TASF) running spirally between the turns which contains both arteries and veins. The CVA enters the cochlea from the internal auditory canal (IAC) and divides into a vestibular and a cochlear branch running in opposite directions.

**Figure 4:**
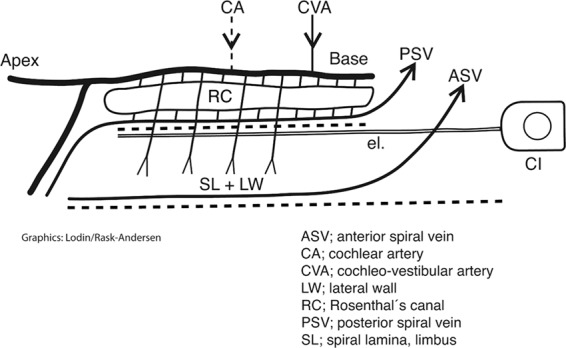
Celloidin section of a human cochlea. (**A**) Framed area shows the modiolus with Rosenthal canal (RC) and several bone channels containing arteries and veins. Framed area is magnified in (**B**). (**B**) The human spiral ganglion (HSG) contains a rich capillary network with arterial feeders derived from superior (arrows). Framed areas C. and D. are magnified in insets. Perilymph fixation and hematoxylin and eosin (H&E) staining were used. Archival section and Innsbruck collection. Celloidin-embedded sections emanated from previous research projects.

A histological section with a corresponding synchrotron projection is shown in Fig. 1A,B, to allow for a direct comparison. The modeled RC consisted of two helical turns with a terminal dilation (Fig. 2A,B). It was smallest near the base and had a flat surface against the perimodiolar wall. Central axons entered inferiorly through several bone channels, while dendrites exited lateral-superiorly into bony columns running to the spiral lamina. The CVA was identified in the IAC (Fig. 3A,B) and could be followed into the cochlea where it divided into a vestibular and a cochlear branch running in opposite directions. The cochlear branch ran apically and anastomosed with several smaller channels. The vestibular branch ran along the inferior border of the most basal part of the RC and saccule, to which it sent vascular twigs. Branches of the CVA joined the spiral modiolar channel assumed to enclose the spiral modiolar artery (SMA) (tractus arteriosus spiralis foraminulentis, or TASF)^5,6^. This channel sent off branches medially and laterally in the lower basal turn. LM showed that these connections contained blood vessels and that the TASF channel contained both arteries and veins (Fig. 4). The spiral ganglion contained rich capillary networks derived from larger vessels located around the RC. They entered via small bony openings but were also together with nerve fascicles located inferiorly and medially. The arterial anatomy was highly variable as previously described by Nabeya^5^. On serial synchrotron X-ray sections from the basal turn, branches of the CVA could be identified at the upper and medial side of the RC, while nerve channels entered inferiorly and medially.

**Figure 5:**
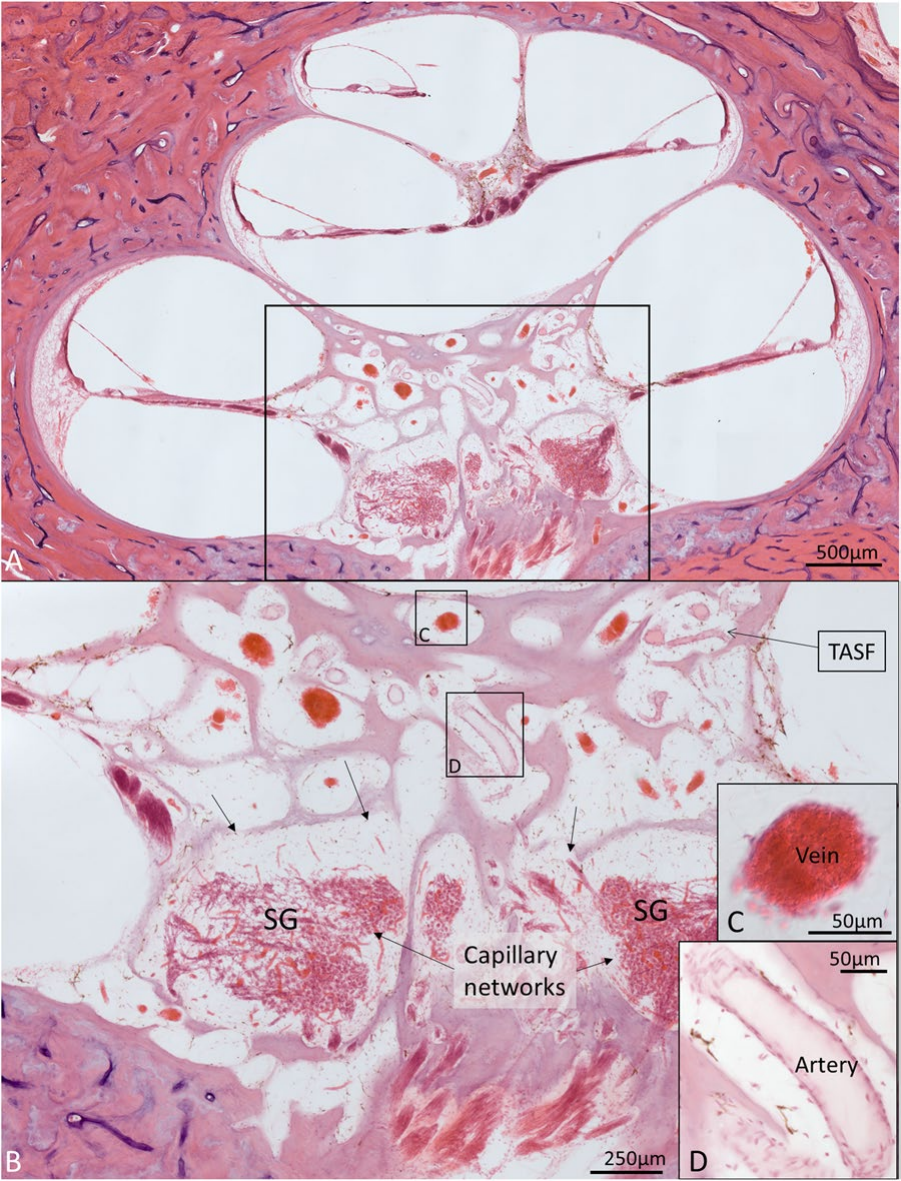
(**A**) Synchrotron radiation phase-contrast imaging (SR-PCI) 3D rendering and segmentation of the tractus arteriosus spiralis foraminulentis (TASF) in the lower basal turn of the cochlea. Radiating arterioles (RAs) are also segmented running from the TASF to the superior wall of the scala vestibuli (SV) at the lower basal turn. (**B**) Same 3D model showing RAs in the upper basal turn running beneath the TASF to the cochlear branch of the cochleo-vestibular artery (CVA). Venules drain blood from the SV into the TASF. (**C**) Scanning electron micrograph shows inner surface of the SV. Venule and arteriole bony openings show different morphology.

Radiating arterioles (RAs) were followed back from the SV (Fig. 5A). At the upper basal turn, surprisingly, these arteries did not always connect to the TASF, but instead joined the cochlear branch of the CVA (Fig. 5B). The CA was not identified in this modeled specimen (1552 R). The CA is otherwise described as entering the modiolus between the lower and upper basal turn of the cochlea, running to the apex, and supplying the entire cochlea, except for the first basal half turn. Axelsson^9^, Nabeya^5^, and Charachon^10^ found that the CA in certain individuals is replaced by the cochlear branch of the CVA supplying all capillary regions. This variation may have clinic-pathological significance, as all cochlear perfusion would be reliant on this single branch. This particular modeled specimen represented such a variation.

### Veins

**Figure 6:**
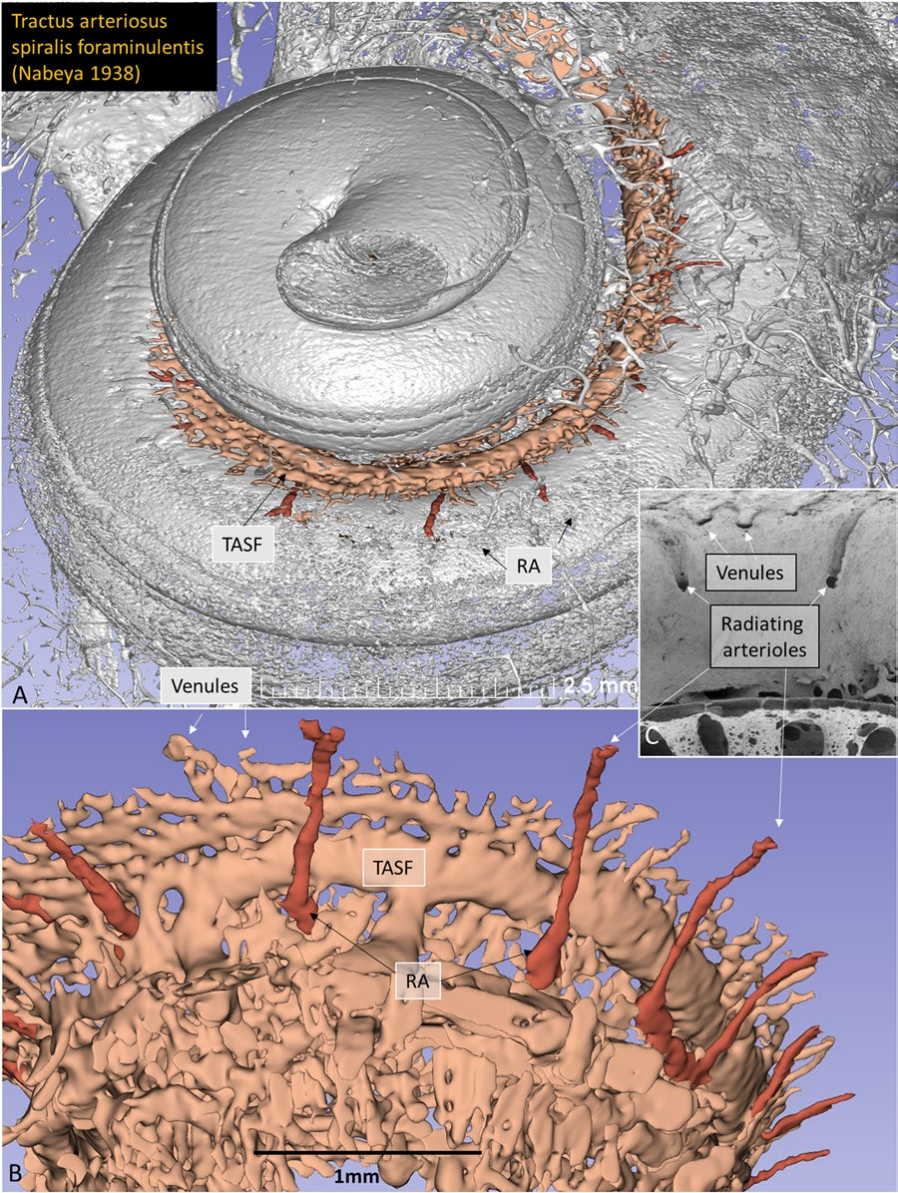
(**A**) Base of the cochlea shown in higher magnification. The cochleo-vestibular artery (CVA) is followed from the internal auditory canal (IAC) to the modiolus. Interrupted lines (**B–E**) correspond to synchrotron sections shown below. B^1^. The human spiral ganglion (HSG) is framed and shown in higher magnification in B^2^. The CVA sends arterial twigs into the RC (**) while veins drain inferiorly (*) directly into the inferior cochlear vein (ICV). The anastomosis between the tractus spiralis arteriosus foraminulentis (TSAF) and the anterior (ASV) and posterior spiral veins (PSV) is seen in Figures. (**C**,**D**) The CVA can be seen in (**D**,**E**) shows the relationship between the TASF and the artery.

**Figure 7:**
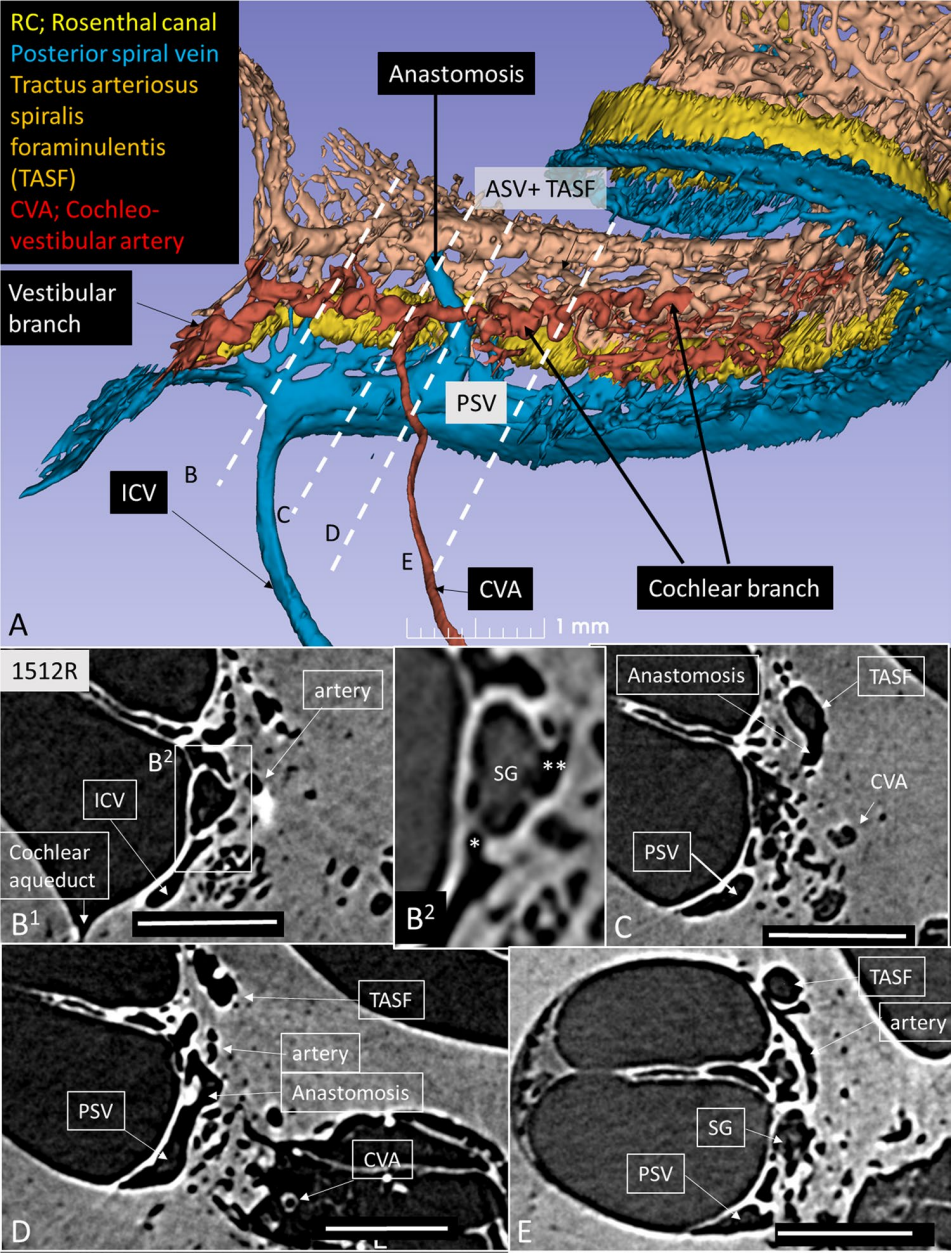
(**A**) Cross-sectioned and 3D rendered contralateral cochlea (1512 L). (**B**) A similar venous anastomosis (*) can be seen between the anterior (ASV) and posterior spiral veins (PSVs) that drain into the inferior cochlear vein (ICV). (**B**) Cross-section of the PSV shows several septum-like structures bridging its lumen. Right arrow shows vein connection to the Rosenthal canal (RC). White arrow shows superior drainage route. TASF = tractus spiralis arteriosus foraminulentis.

Our findings verified the results of Nabeya^5^ and Axelsson^9^, indicating that the venous drainage is complex and consists of two systems: the PSV, which approaches the infero-medial wall of the ST, and the ASV, which drains blood from the SV, spiral lamina, and modiolus (Fig. 6). We found that these pathways varied significantly. In our model, both pathways contributed considerably to the venous outflow and united at the base of the cochlea before emptying into the ICV. Surprisingly, connections existed between the ASV and PSV in the right modeled ear (Fig. 6) and also the contralateral left ear where the ASV directly emptied into the ICV (Fig. 7). SR-PCI sections and 3D reconstruction with fiducial markers verified the connection between the ASV and PSV (Fig. 6). The ASV collected blood from the SV and coursed into the TASF channel. Multiple collecting veins drained into the PSV in the ST in the basal turn of the cochlea. It was not possible to follow the venous twigs in detail in the apical coil. The PSV also collected blood from the lateral wall of the cochlea, the modiolus, and spiral ganglion, as well as from the vein of the round window (RW) and vestibule (Fig. 7). Orthogonal sectioning and retrograde tracing in the basal coil identified many venous openings into the modiolus from the RC (Fig. 7 inset). The PSV contained septum-like pillars, presumably to remain open under low pressure conditions.

## Discussion

This study presents the first 3D segmentation and analysis of the HSG blood supply. SR-PCI, 3D rendering, and modeling made it possible to better evaluate the contributions of major- and medium-sized vessels to the human RC. Through LM the capillary networks were able to be evaluated.

The 3D reconstructions and histological sectioning showed that the envelope of the TASF described by Siebenmann^6^ and Nabeya^5^ may not act solely as a major arterial contributor to the cochlea. Nabeya described it as a very long and powerful branch which occupies the blood vessels of the whole cochlea. The bone channel also contains the ASV, and the conditions in this model may adhere to the second kind of arterial supply described by Nabeya^5^. The arterial supply was derived primarily from the cochlear branch of the CVA. The prominent CVA was connected to the TASF at several locations and supplied the second and third turns of the cochlea. The RAs supplying the lateral wall beyond the lower basal turn did not originate from the TASF, but from the CVA in this specimen. The CA (not verified here) is otherwise the main branch of the labyrinthine artery and reaches the cochlea after half a turn^5^. Axelsson^9^ found that, when the CA is replaced by the cochlear branch of the CVA, individuals may be more vulnerable to vascular conditions in the cochlea. Again, the anatomic variability is wide, and in other investigated bones, the CVA and the cochlear branch ran against the apex to anastomose with the CA via the TASF. This represents the first type of arterial supply which was described by Nabeya^5^.

**Figure 8:**
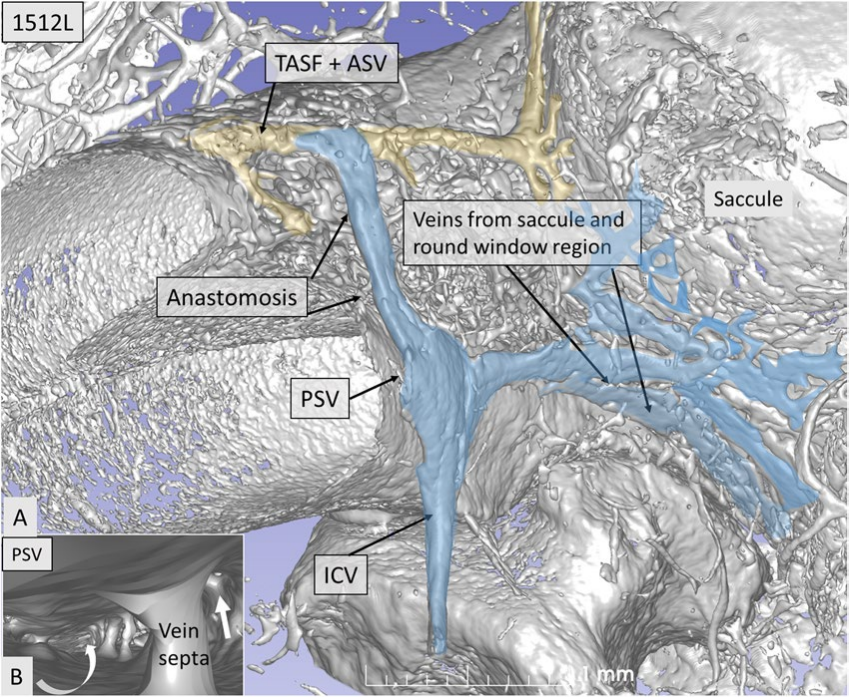
Schematic illustration of the principal vascular supply of the human spiral ganglion (HSG) and its relevance for cochlear implantation (CI) function. Unlike most organs, arteries and veins in the cochlea do not follow the same conduits. The HSG cells in the Rosenthal canal (RC) are supplied with arteries derived from several branches from the cochlea-vestibular artery (CVA) and the cochlear artery (CA). There is also a dual venous outflow via the anterior spiral vein (ASV) and posterior spiral vein (PSV). Thereby, the nerve cells are “circulatory” and protected from the rest of the cochlea. Their relative independence may explain nerve cells frequent resistance to undergo degeneration in connection with cochlear pathology essential at CI.

The ganglion cell bodies are located in a fenestrated bony enclave containing approximately 35,000 neurons^22,30^. It extends as a 13–14 mm long helix^31^ with segments richly supplied by radial tributaries from larger vessels around the bony wall. Contributing vessels are sheltered in bone, and the draining ICV is shielded partly by a thin sheet of bone in the floor of the ST. A schematic of the vascular supply of the HSG is shown in Fig. 8. Multiple radiating vessels to and from the RC may secure the vascular supply and its relative independence from the rest of the cochlea. The rich segmental supply and double drainage systems may explain the resistance of the HSG to undergo degeneration despite extensive cochlear pathology. There is also a documented slow retrograde degeneration of the human cochlear nerve following hair cell loss^32,33^ that may be beneficial for cochlear implantation^34^. These conditions may contribute to the fact that CI recipients, even with “totally deaf” ears, may still have a functional nerve reservoir suitable for electric stimulation. Unlike the protected arterial supply, venous outflow may be susceptible to damage during CI insertion. In the current study, the venous outflow was covered by a thin ledge of bone near its exit in the floor of the ST. Therefore, even though experimental studies show that arterial obstruction causes more severe damage than obstruction of the ICV^35^, surgical procedures such as drilling at the floor of the RW niche or extended RW approaches could lead to damage of the ICV^36^. In a recent study by Atturo *et al*.^36^ using µCT and computer-based 3D reconstructions with virtual sectioning, damage to the ICV was considered unlikely in most cases at CI drilling, although at some anatomic variations, surgery could challenge venous outflow and neuron survival.

In the present study, small capillaries and individual nerve fibers were difficult to visualize. Therefore, further studies are required to improve soft tissue resolution of the intact human cochlea by modifications in SR-PCI imaging, including sample preparation and image acquisition.

## Conclusions

This study presents the first 3D segmentation and analysis of the HSG blood supply. The HSG is supplied by radial vascular twigs which are separate from the rest of the inner ear and encased in bone. This, together with the slow retrograde nerve degeneration, may explain the HSG’s viability in CI recipients, even in advanced cochlear pathology. Venous outflow may also be susceptible to damage during CI insertion.
