# Publisher Correction: Vascular Supply of the Human Spiral Ganglion: Novel Three-Dimensional Analysis Using Synchrotron Phase-Contrast Imaging and Histology

**DOI:** 10.1038/s41598-020-64611-2

**Published:** 2020-05-01

**Authors:** Xueshuang Mei, Rudolf Glueckert, Annelies Schrott-Fischer, Hao Li, Hanif M. Ladak, Sumit K. Agrawal, Helge Rask-Andersen

**Affiliations:** 1 Department of Surgical Sciences, Section of Otolaryngology, Uppsala University Hospital, SE, 751 85 Uppsala, Sweden; 2 Department of Otolaryngology, Peking University Shenzhen Hospital, Shenzhen, China; 3 Department of Otolaryngology, Medical University of Innsbruck, Anichstr. 35, A-6020 Innsbruck, Austria; 4 Department of Otolaryngology-Head and Neck Surgery, Department of Medical Biophysics and Department of Electrical and Computer Engineering, Western University, London, ON Canada; 5 Department of Otolaryngology-Head and Neck Surgery, Western University, London, ON Canada

Correction to: *Scientific Reports* 10.1038/s41598-020-62653-0, published online 03 April 2020

This Article contains an error in the order of the Figures. Figure 8 was published as Figure 4 and Figures 4, 5, 6 and 7 were published as Figures 5, 6, 7 and 8 respectively. The correct Figures 4, 5, 6, 7 and 8 appear below as Figures 1, 2, 3, 4 and 5. The Figure legends are correct.

**Figure 1:**
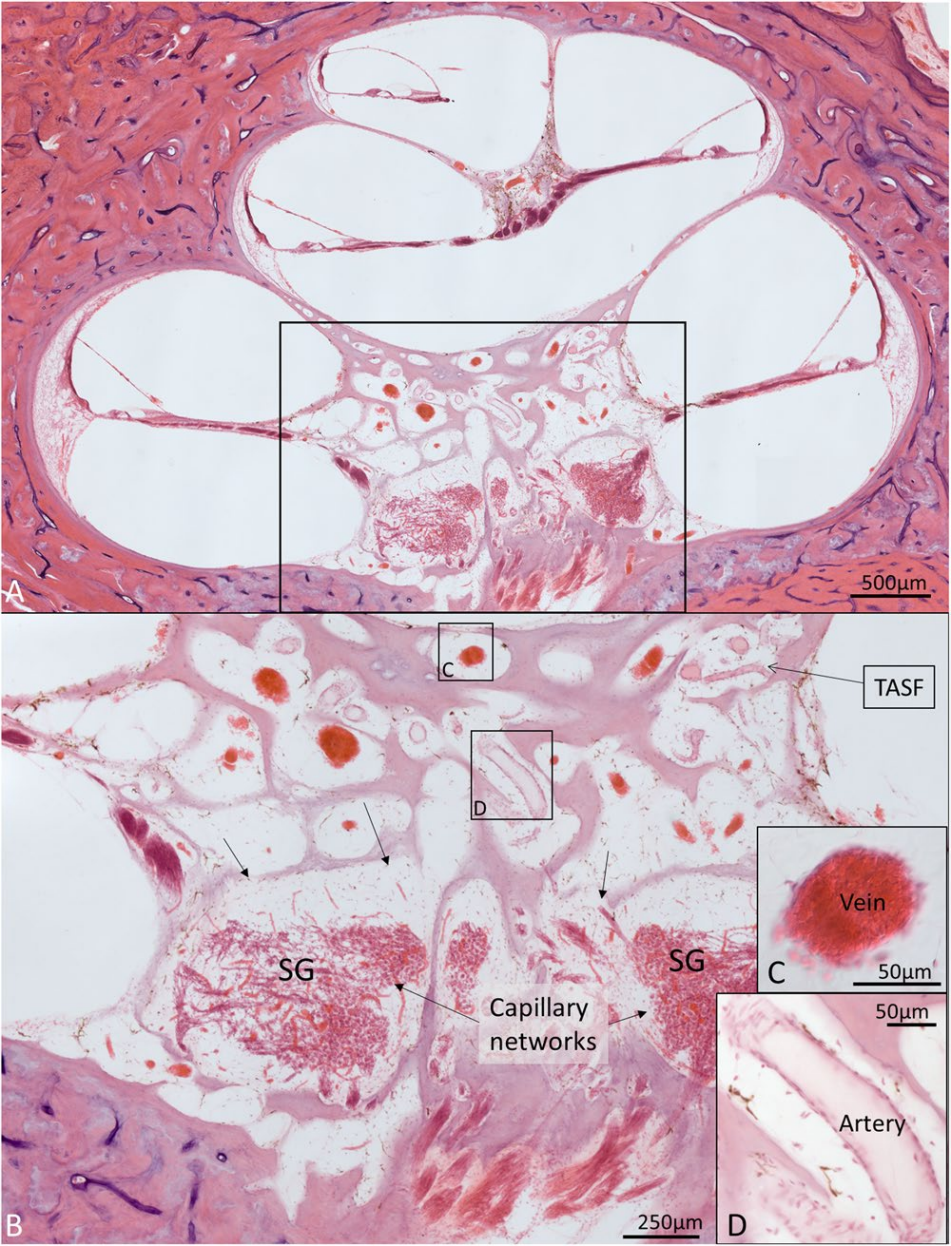
.

**Figure 2:**
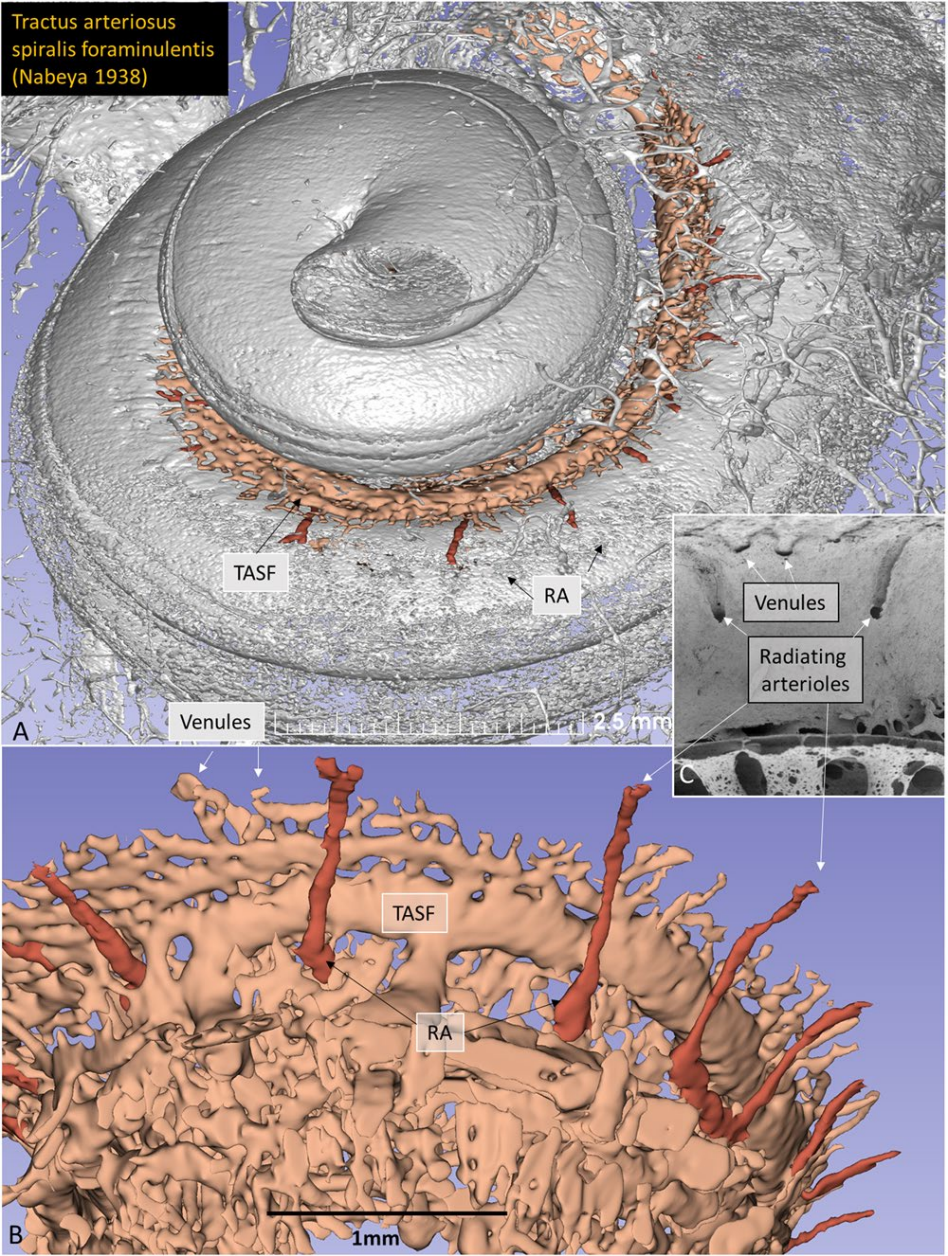
.

**Figure 3:**
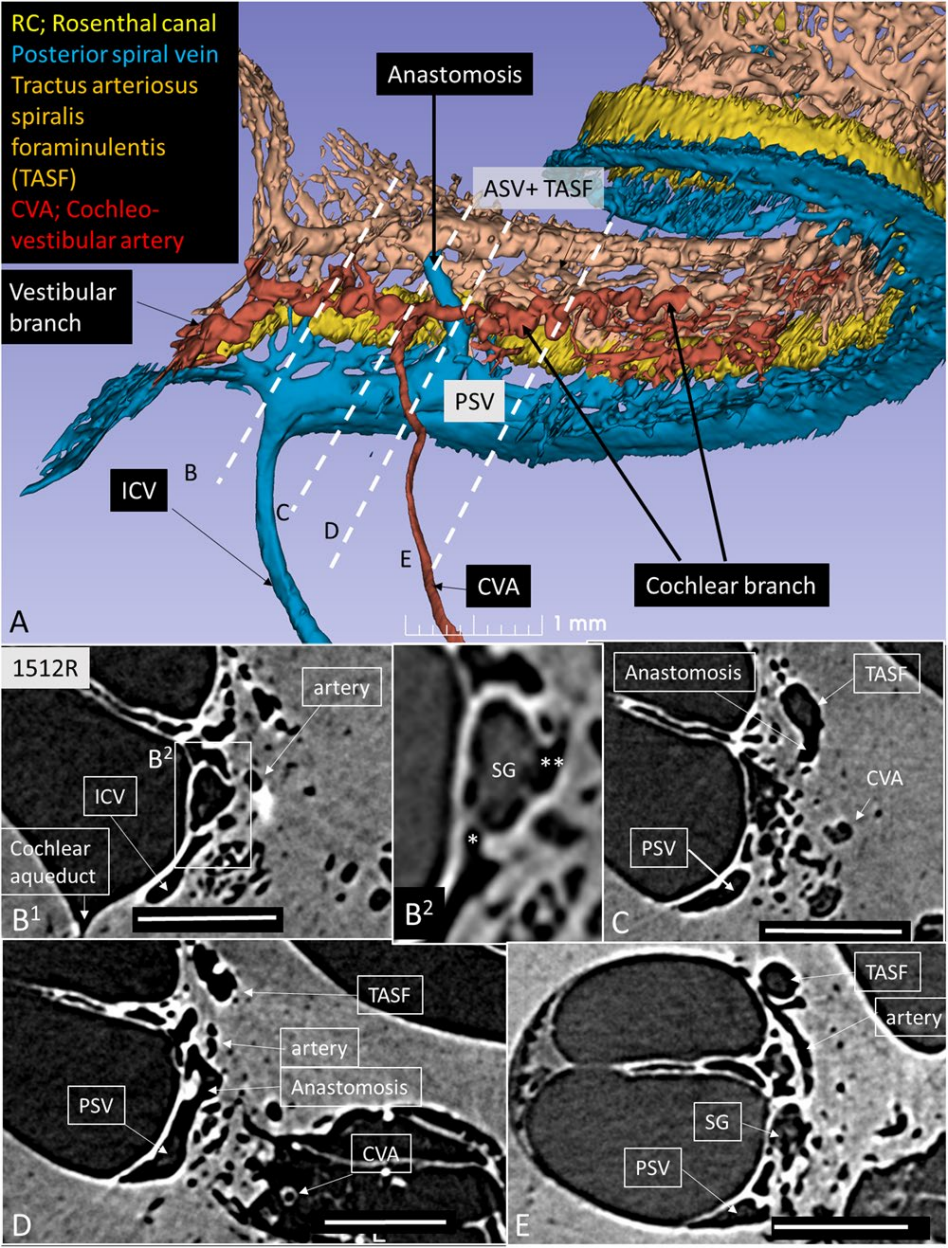
.

**Figure 4:**
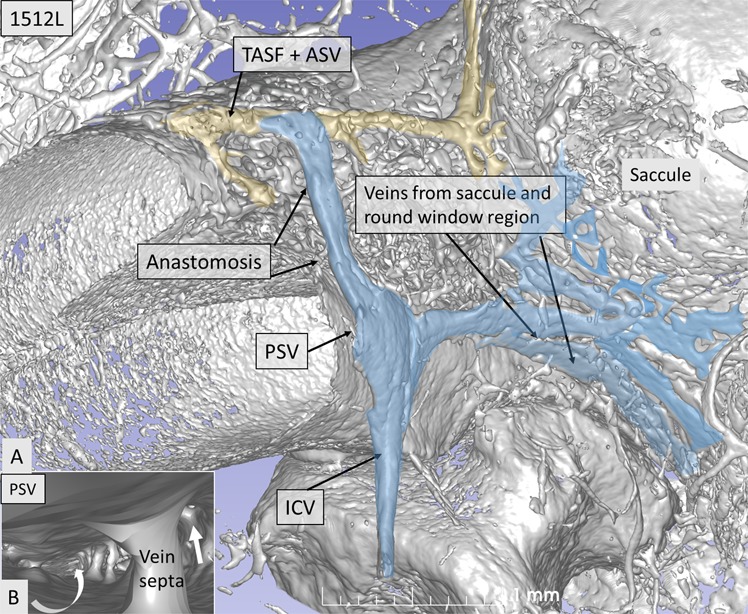
.

**Figure 5:**
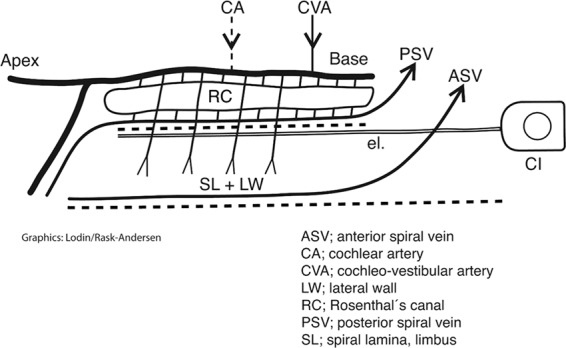
.

